# Supplementary material for: Key features of the genetic architecture and evolution of host-microbe interactions revealed by high-resolution genetic mapping of the mucosa-associated gut microbiome in hybrid mice
Source: eLife. 2022 Jul 19;11:e75419. doi: 10.7554/eLife.75419 (PMC9307277; doi:10.7554/eLife.75419)
Supplement: Transparent reporting form [file elife-75419-transrepform1.docx]

***eLife’s* transparent reporting form**

We encourage authors to provide detailed information *within their submission* to facilitate the interpretation and replication of experiments. Authors can upload supporting documentation to indicate the use of appropriate reporting guidelines for health-related research (see [EQUATOR Network](about:blank)), life science research (see the BioSharing Information Resource), or the [ARRIVE guidelines](about:blank) for reporting work involving animal research. Where applicable, authors should refer to any relevant reporting standards documents in this form.

If you have any questions, please consult our Journal Policies and/or contact us: [editorial@elifesciences.org](about:blank).

**Sample-size estimation**

- You should state whether an appropriate sample size was computed when the study was being designed
- You should state the statistical method of sample size computation and any required assumptions
- If no explicit power analysis was used, you should describe how you decided what sample (replicate) size (number) to use

Please outline where this information can be found within the submission (e.g., sections or figure legends), or explain why this information doesn’t apply to your submission:

Lines 536-537. For power analysis, we simulated F1 and F2 generations from phased SNP data (GigaMUGA array data) using the R package *simcross* and custom perl scripts. Simulations were performed with different phenotypic variance estimates (5%, 10%, 20%) for 10 different phenotypes. Each simulation was performed for 150 and 300 (large data set) individuals and for an 8-way cross design. Afterwards, QTL analysis were performed to retrieve mapping intervals (in cM) and LOD scores for each simulated QTL. In all analysis, the large data set showed smaller mapping intervals and higher LOD scores for causal SNPs.

For a phenotypic variance of 5%, 73.8% of the QTLs were detected in the simulation. For increasing phenotypic variance (10%, 20%), this value increased to 87.5%.

A similar analysis was performed for the 8-way cross, using 38 individuals per cross (304 mice in total). Here, 66% of the QTLs were detected if the phenotypic variance is low (5%), 94% were detected when the phenotypic variance was intermediate (10%) and 100% were detected for high phenotypic variance (20%).

**Replicates**

- You should report how often each experiment was performed
- You should include a definition of biological versus technical replication
- The data obtained should be provided and sufficient information should be provided to indicate the number of independent biological and/or technical replicates
- If you encountered any outliers, you should describe how these were handled
- Criteria for exclusion/inclusion of data should be clearly stated
- High-throughput sequence data should be uploaded before submission, with a private link for reviewers provided (these are available from both GEO and ArrayExpress)

Please outline where this information can be found within the submission (e.g., sections or figure legends), or explain why this information doesn’t apply to your submission:

Excluded samples where phenotyping failed: lines 575-577

Reviewer link sequence data: [https://dataview.ncbi.nlm.nih.gov/object/PRJNA759194?reviewer=7c8p1dsv7pt117lcbc7r26kfs0](about:blank)

**Statistical reporting**

- Statistical analysis methods should be described and justified
- Raw data should be presented in figures whenever informative to do so (typically when N per group is less than 10)
- For each experiment, you should identify the statistical tests used, exact values of N, definitions of center, methods of multiple test correction, and dispersion and precision measures (e.g., mean, median, SD, SEM, confidence intervals; and, for the major substantive results, a measure of effect size (e.g., Pearson's r, Cohen's d)
- Report exact p-values wherever possible alongside the summary statistics and 95% confidence intervals. These should be reported for all key questions and not only when the p-value is less than 0.05.

Please outline where this information can be found within the submission (e.g., sections or figure legends), or explain why this information doesn’t apply to your submission:

(For large datasets, or papers with a very large number of statistical tests, you may upload a single table file with tests, Ns, etc., with reference to sections in the manuscript.)

| **Reference** | **Statistical test** |
| --- | --- |
| Correlation between host genetic relatedness and microbiome structure: lines 136, 610-620 | Mantel test with Spearman’s correlation with 10,000 permutations |
| SNP-based heritability: 142, 621-629, Fig 1A-B, Suppl. Table 1 | Restricted Log-Likelihood test (RLRT) |
| SNP-based heritability: 150, Suppl. Fig 4 | Spearman’s correlation |
| Heritability estimates are correlated with predicted co-speciation rates: 161-162, Fig 1C-D | Spearman’s correlation |
| Genetic mapping of host loci determining microbiome composition: 175-182, Table 1, Suppl. Table 2, 630-672 | Linear mixed model |
| Functional annotation of candidate genes: 266, 689-702 | STRING PPI enrichment test |
| Functional annotation of candidate genes: 279-287, 703-706 | One-tailed Fisher Exact test, BH correction for multiple testing |
| Functional annotation of candidate genes: 293-295, 707-713 | Chi-squared |
| Comparison of significant loci to published gut microbiome mapping studies: 312-313, 322-323, 707-713 | Chi-squared |
| Proteins differentially expressed in GF vs conventional mice: 339-340, 707-713 | Chi-squared |
| Proteins differentially expressed in GF vs conventional mice: 341-342 | STRING PPI enrichment test |
| Candidate genes influencing bacterial abundance: 352-354 | STRING PPI enrichment test |

**Group allocation**

- Indicate how samples were allocated into experimental groups (in the case of clinical studies, please specify allocation to treatment method); if randomization was used, please also state if restricted randomization was applied
- Indicate if masking was used during group allocation, data collection and/or data analysis

Please outline where this information can be found within the submission (e.g., sections or figure legends), or explain why this information doesn’t apply to your submission:

We do not have different experimental groups.

**Additional data files (“source data”)**

- We encourage you to upload relevant additional data files, such as numerical data that are represented as a graph in a figure, or as a summary table
- Where provided, these should be in the most useful format, and they can be uploaded as “Source data” files linked to a main figure or table
- Include model definition files including the full list of parameters used
- Include code used for data analysis (e.g., R, MatLab)
- Avoid stating that data files are “available upon request”

Please indicate the figures or tables for which source data files have been provided:

All the scripts used for performing data analysis and visualisation are available at [https://github.com/sdoms/mapping_scripts](about:blank), including the genotype and phenotype data files, allowing all figures and tables to be reproduced.

In addition:
Fig 1: heritability estimates provided as Suppl. Table 1

Fig 2, Table 1: Genome-wide significant associations provided as Suppl. Table 2, Study-wide significant associations provided as Suppl. Table 3

Fig 6: Candidate genes provided in Suppl. Table 7
